# Trajectories of physical activity from midlife to old age and associations with subsequent cardiovascular disease and all-cause mortality

**DOI:** 10.1136/jech-2019-212706

**Published:** 2019-11-08

**Authors:** Daniel Aggio, Efstathios Papachristou, Olia Papacosta, Lucy T Lennon, Sarah Ash, Peter Whincup, S Goya Wannamethee, Barbara J Jefferis

**Affiliations:** 1 Primary Care and Population Health, University College London, London, UK; 2 Department of Psychology & Human Development, University College London, London, UK; 3 Population Health Research Institute, St George’s University of London, London, UK

**Keywords:** ageing, cardiovascular disease, CHD/coronorary heart, life course epidemiology, physical activity

## Abstract

**Introduction:**

It is well established that physical activity (PA) protects against mortality and morbidity, but how long-term patterns of PA are associated with mortality and cardiovascular disease (CVD) remains unclear.

**Methods:**

3231 men recruited to the British Regional Heart Study, a prospective cohort study, reported usual PA levels at baseline in 1978–1980 (aged 40–59 years) and at 12-year, 16-year and 20-year follow ups. Twenty-year trajectories of PA, spanning from 1978/1980 to 2000, were identified using group-based trajectory modelling. Men were subsequently followed up until 30 June 2016 for mortality through National Health Service central registers and for non-fatal CVD events through primary and secondary care records. Data analyses were conducted in 2019.

**Results:**

Three PA trajectories were identified: low/decreasing (22.7%), light/stable (51.0%) and moderate/increasing (26.3%). Over a median follow-up of 16.4 years, there were 1735 deaths. Compared with the low/decreasing group, membership of the light/stable (HR 0.83, 95% CI 0.74 to 0.94) and moderate/increasing (HR 0.76, 95% CI 0.66 to 0.88) groups was associated with a lower risk of all-cause mortality. Similar associations were observed for CVD mortality, major coronary heart disease and all CVD events. Associations were only partially explained by a range of confounders. Sensitivity analyses suggested that survival benefits were largely driven by most recent/current PA.

**Conclusions:**

A dose-response relationship was observed, with higher levels of PA from midlife to old age associated with additional benefits. However, even fairly modest and sustained PA was protective and may be more achievable for the most inactive.

## Introduction

Physical activity (PA) is inversely associated with risk of all-cause mortality and cardiovascular disease (CVD) events in old age.^1–4^ Most of what is known about this relationship is based on studies using single measures of PA.^5–8^ However, major life transitions such as retirement are periods when PA may be sensitive to change.^9–11^ Previous studies suggest that initiation or maintenance of an active lifestyle can reduce mortality risks^4 12–18^ but the majority of these studies capture change across two time points only. Utilising additional repeated measures may give a more reliable estimate of the relationship between long-term PA and mortality.

Furthermore, conventional methods for defining PA changes are theory driven, which typically involves dividing subjects into clinically meaningful groups based on a relevant cut point (ie, maintainers, adopters, drop outs).^19 20^ However, this approach relies on the assumption that these patterns truly exist. By contrast, data-driven approaches, such as group-based trajectory modelling (GBTM), allow the most naturally occurring trajectories to emerge from the data rather than assuming predetermined trajectories.^21^ This method has been used to identify PA trajectories in older adults,^22–25^ but associations with subsequent mortality and CVD events have not been fully explored.

Two previous studies using GBTM suggested that more active trajectories are associated with a reduced risk of all-cause, cancer-related and CVD-related mortality.^25 26^ However, trajectories were either retrospectively reported^26^ or focused on a fairly short period of the adult life course.^25^ These findings have yet to be replicated with longer prospective follow-up for PA, while also adjusting for established and novel CVD biomarkers. Associations with CVD subtypes also remain unclear. This study aimed to identify 20-year PA trajectories from midlife to old age using GBTM and to examine associations with subsequent major coronary heart disease (CHD) and stroke events and all-cause and CVD mortality.

## Methods

### Participants

The British Regional Heart Study (BRHS) is a prospective cohort study involving 7735 men (aged 40–59 years at baseline) recruited from primary care practices in 24 towns in Great Britain between 1978 and 1980.^27^ Men completed a lifestyle and medical history questionnaire at baseline, 12-year, 16-year and 20-year follow ups and attended physical examinations at baseline and the 20-year follow-up. Data collection spanned 1978–2016, and data were analysed in 2019.

### Measurements

### Pre-existing disease and CVD risk factors

At the 20-year follow-up, participants attending the physical examination provided a fasting blood sample, which was analysed for total, high-density lipoprotein (HDL) and low-density lipoprotein cholesterol (LDL), insulin, von Willebrand factor (vWf), interleukin 6 (IL-6), n-terminal pro-brain natriuretic peptide (NT-proBNP) and high-sensitivity cardiac troponin T (Hs-TnT). Other physical measurements including waist circumference, blood pressure and lung function (forced expiratory volume in one second (FEV_1_)) were taken by nurses. Information on measurement techniques are described in the supplementary files (online supplementary text A). Men also reported whether they had ever been diagnosed with a heart attack (coronary thrombosis or myocardial infarction (MI)), stroke or diabetes. Fasting blood glucose measures of ≥7 mmol/L confirmed diabetes diagnosis. Men also self-reported employment status (employed or not in employment); current or longest held occupation (manual or non-manual); marital status (single, married or widowed/divorced); number of children (none or ≥1); doctor-diagnosed health conditions (arthritis, bronchitis and high blood pressure); other health problems (breathlessness and chest pain on exertion); smoking status (current/recent ex-smoker or non-smokers/long-term ex-smoker (>15 years)), alcohol consumption (none, occasional (<1 drink/week), light (1–15 drinks/week), moderate (16–42 drinks/week) or heavy (>42 drinks/week)); region of residence (Scotland, North, Midlands and South) and breakfast cereal consumption (none, occasional (1–2 times/week) or regular (>3 times/week)). Baseline body mass index (BMI) was derived from height and weight measurements (normal weight: BMI <25.0 kg/m^2^ or overweight/obese: BMI ≥25.0 kg/m^2^).

10.1136/jech-2019-212706.supp1 Supplementary data

### Self-reported PA

At baseline, 12-year, 16-year and 20-year follow ups, participants reported their usual PA, including time spent on all forms of walking, recreational activities (such as recreational walking, gardening, chores and do-it-yourself activities) and sport/exercise. Questions and response options were virtually identical at each wave. Responses were scored based on the intensity and frequency of the activity.^28 29^ A total PA index was calculated by summing the scores for each item. The original scoring system has been reported elsewhere.^30^ The total PA index was then collapsed into a six-point score (0–5); inactive (0); occasional (1) (regular walking or recreational activity only); light (2) (more-frequent recreational activities, sporting exercise less than once a week, or regular walking plus some recreational activity); moderate (3) (cycling, very frequent weekend recreational activities plus regular walking, or sporting activity once a week); moderately vigorous (4) (sporting activity at least once a week or frequent cycling, plus frequent recreational activities or walking, or frequent sporting activities only); or vigorous (5) (very frequent sporting exercise or frequent sporting exercise plus other recreational activities). The PA score has been validated against heart rate, FEV_1_ 30 and device-measured PA.^31^

### Mortality and morbidity

Men were followed up for major CVD events and mortality from any cause from the 20-year follow-up in 2000 until 30 June 2016. Information on cause of death was collected through the tagging procedures of the National Health Service Central Registers in Southport for England and Wales, and in Edinburgh for Scotland (death certificates coded using the International Classification of Diseases, Ninth Revision (ICD-9)). Information on non-fatal events was obtained from general practitioners and biennial reviews of the patients’ medical records. For major CHD events (fatal/non-fatal), fatal MI was defined as ICD-9 codes 410–414 and non-fatal MI was defined as heart attack or coronary thrombosis in accordance with the WHO diagnostic criteria. For major stoke events (fatal/non-fatal), fatal stroke was defined as ICD-9 codes 430–438 and non-fatal stroke events included those that caused a neurological deficit for >24 hours. Fatal CVD was defined as ICD-9 codes 390–459. Outcome measures included all-cause and CVD mortality, major CHD events (fatal/non-fatal), major stroke events (fatal/non-fatal) and all CVD events (fatal/non-fatal).

### Statistical analysis

### Identifying PA trajectory groups

GBTM was used to identify 20-year PA trajectories over the four waves. Models were conducted using the Stata TRAJ plugin,^32^ which applies finite mixture modelling and maximum likelihood estimation to identify latent groups of individuals that follow similar trajectories. Models with 2–5 trajectory groups were tested and compared using goodness-of-fit criteria. The best fitting model was determined based on the highest (ie, least negative) Bayesian information criterion, the log Bayes Factor (2*Δ Bayesian information criterion), trajectory group sizes of >5%, close agreement between estimated and actual trajectory group prevalence, posterior probabilities >0.70 and odds of correct classification based on posterior probabilities exceeding 5.^21^ Subjects were assigned to the group that they had the highest probability of belonging to. Models simultaneously incorporated baseline predictors including age, occupational class, marital status, number of children, region of residence, diagnosed health conditions (arthritis, bronchitis and high blood pressure), BMI, smoking status, alcohol consumption and breakfast cereal consumption as well as time-varying variables, including number of CVD events and employment status. Trajectory shapes were determined by reducing the level of the polynomial function, starting with quadratic, until each growth parameter estimate was statistically significant (p<0.05). Descriptive statistics for demographic, lifestyle and CVD risk factors are presented according to trajectory groups.

### Survival analysis

Cox proportional hazards models were used to estimate associations between trajectory groups and all-cause and CVD mortality, major CHD, stroke and all CVD events. For participants with no events, data were censored on the 30 June 2016. The proportional hazards assumption was examined using graphical methods and Schoenfeld residuals. Model 1 adjusted for age, marital status, alcohol consumption, smoking status and previous diagnosis of MI, stroke and diabetes (reported at the 20-year follow-up), and occupational class and region (reported at baseline). To examine the role of potential mediators, model 2 additionally adjusted for metabolic CVD risk factors measured at the 20-year follow-up, including LDL, HDL, systolic blood pressure, insulin, waist circumference and FEV_1_. Model 3 additionally adjusted for the inflammatory and haemostatic markers IL-6 and vWf. Model 4 additionally adjusted for the cardiac markers Hs-TnT and NT-proBNP.

### Additional analysis

As CVD events can influence PA and increase the risk of mortality, trajectories were also identified after excluding participants with CVD at the 20-year follow-up and associations with subsequent outcomes were estimated. Furthermore, as undiagnosed chronic conditions, such as CVD (eg, heart failure), may initiate changes in PA and increase the risk of mortality, analyses were performed after excluding the first 2 years of follow-up, thus minimising the possibility of reverse causation. As well as using GBTM to identify trajectories, we also grouped subjects based on observed trajectories using binary exposure measurements from baseline through 12-year and 20-year follow ups. The PA score was collapsed into a binary variable at each of the three time points, with 1 indicating at least ‘light’ PA levels and 0 denoting ‘inactive’ or ‘occasional’ activity levels. This cut point has previously been used in studies using data from this cohort^16 33^ and was informed by a recent validation study, which highlighted a large increment in PA between those who are classified as ‘inactive’ or ‘occasionally active’ and other PA scores.^31^ There are eight possible trajectories indicated by combinations of zero and one. For example, (0-0-0) represents low PA at all periods, while (1-0-0) indicates high PA at baseline only. This approach for classifying trajectories can identify sensitive periods when exposures during specific life stages might have a particularly strong effect on subsequent outcomes.^34^ Analyses were performed on selected outcomes with these alternate trajectory groupings.

## Results

From the 7735 men recruited at baseline, 5516 survived the next 20 years of follow-up, of which 4252 attended the 20-year physical examination (77% of survivors). Of these, 197 men with <3 PA measures were excluded and a further 824 men were excluded due to missing covariate data, leaving 3231 available for analyses. Compared with the final sample, excluded men (n=4504) were significantly older at baseline (52.4 vs 48.7 years; p<0.001), were more likely to be inactive (43.3% vs 34.9%; p<0.001), come from manual occupations (66.3% vs 52.0%; p<0.001) and suffer from a range of health conditions, including breathlessness (9.3% vs 2.8%; p<0.001), overweight/obesity (55.3% vs 52.7%; p=0.020), arthritis (11.6% vs 8.1%; p<0.001) and bronchitis (20.8% vs 14.2%, p<0.001).

**Figure 1:**
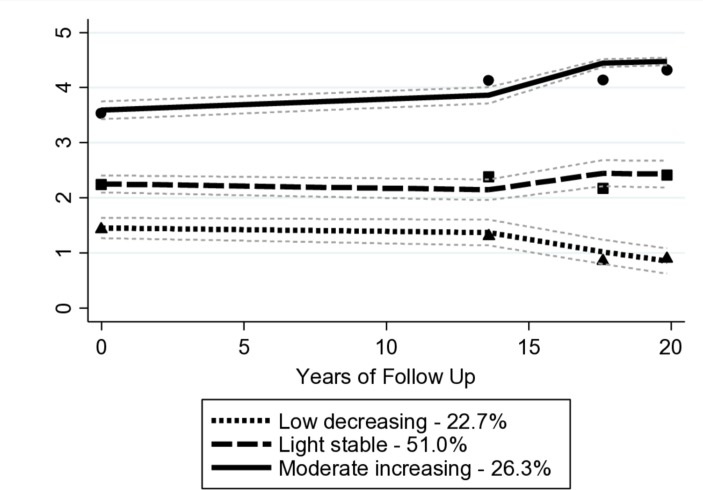
Group-based trajectory modelling-derived physical activity trajectories and 95% CI from midlife to old age (n=3231).

Three trajectory groups emerged as the most parsimonious description of the longitudinal data: low-decreasing (22.7%), light-stable (51.0%) and moderate-increasing (26.3%) (see figure 1). Results from the model selection process are provided in supplementary files, see online supplementary tables S1 and S2. A dose–response relationship was observed across trajectory groups, whereby men in the low-decreasing trajectory group were older, more likely to come from manual occupations, smoke, come from regions other than the south of England, suffer from a range of health conditions and have a worse cardiovascular risk profile (table 1).

**Table 1:** Subject characteristics at 20 year follow-up according to GBTM physical activity trajectories (n=3231)

Characteristic	Low decreasing (n=733)	Light stable (n=1649)	Moderate increasing (n=849)	All (n=3231)
Age, years (mean ± SD)	69.4 (5.6)	68.5 (5.6)	67.8 (5.1)	68.5 (5.5)
Manual occupation (%, n)*	59.8 (438)	55.6 (916)	38.5 (327)	52.0 (1681)
Alcohol consumption, ≥light† (%, n)	54.6 (400)	62.4 (1029)	73.5 (591)	63.5 (1178)
Current smoker (%, n)	19.5 (143)	10.6 (174)	7.4 (63)	11.8 (380)
Married (%, n)	80.5 (590)	85.0 (1402)	86.9 (738)	84.5 (2730)
Resident in southern England (%, n)*	26.2 (192)	36.2 (597)	38.2 (324)	34.5 (1113)
Waist circumference, cm (mean±SD)	100.1 (11.2)	96.4 (10.0)	95.4 (9.5)	97.0 (10.3)
LDL, mmol/L (mean±SD)	3.8 (1.0)	4.0 (1.0)	3.9 (0.9)	3.9 (1.0)
HDL, mmol/L (mean±SD)	1.3 (0.3)	1.3 (0.3)	1.4 (0.3)	1.3 (0.3)
Insulin, μ/mL (median, IQR)	9.6 (8.1)	8.1 (5.9)	7.5 (5.4)	8.2 (6.4)
SBP, mm Hg (mean±SD)	148.6 (23.9)	149.0 (23.8)	148.0 (24.4)	148.6 (24.0)
FEV_1_, L‡ (mean±SD)	235.2 (69.5)	259.6 (62.5)	276.1 (61.7)	258.4 (65.5)
IL-6, pg/mL (median, IQR)	2.8 (2.4)	2.2 (1.8)	2.0 (1.5)	2.2 (1.9)
vWf, IU/dL (mean±SD)	148.7 (48.4)	137.1 (45.0)	133.6 (43.0)	138.8 (45.6)
Hs-TnT, pg/mL (median, IQR)	12.7 (8.1)	11.4 (7.0)	11.5 (6.3)	11.7 (7.0)
NT-proBNP, pg/mL (median, IQR)	112.0 (206.0)	88.0 (136.0)	78.0 (111.0)	89.0 (144)
Previous MI (%, n)	9.1 (67)	7.3 (120)	6.0 (51)	7.4 (238)
Previous stroke (%, n)	3.3 (24)	2.4 (39)	2.2 (19)	2.5 (82)
Previous diabetes (%, n)	14.5 (106)	7.4 (122)	7.0 (59)	8.9 (287)

*Reported at baseline.

†light classified as ≥1 unit per week.

‡Standardised for height by multiplying FEV_1_ by the square of the mean population height (metres) divided by each participant’s height.

FEV_1_, forced expiratory volume in 1 s;GBTM, group-based trajectory modelling; HDL, high-density lipoprotein; Hs-TnT, high-sensitivity cardiac troponin T; IL-6, interleukin 6; LDL, low-density lipoprotein; MI, myocardial infarction; NT-proBNP, N-terminal pro-brain natriuretic peptide; SBP, systolic blood pressure; vWf, von Willebrand factor.

Over a median follow-up of 16.4 years, there were 1735 deaths. As shown in table 2, unadjusted mortality rates were lowest for the moderate-increasing trajectory group (30.9 per 1000 person-years) and highest for the low-decreasing group (58.8 per 1000 person-years). Compared with the low-decreasing group, membership of the light-stable and moderate-increasing trajectory groups was associated with a lower risk of all-cause mortality in minimally adjusted models. This association was somewhat attenuated after adjusting for cardiometabolic, inflammatory and cardiac biomarkers but remained statistically significant. There was a significant linear trend across trajectory groups, suggesting added benefit for higher PA volumes. Similar patterns were observed for CVD events, CVD mortality and major CHD events, but trajectories were not associated with major stroke events. Additional analysis showed a significant risk reduction in fatal but not non-fatal stroke in men following a light-stable trajectory compared with the low-decreasing group (data not shown).

**Table 2:** Association between physical activity trajectories and subsequent risk of CVD events and all-cause and CVD mortality (n=3231)

Outcome	Trajectory group	N	No. of events	Person-years	Rate per 1000 person-years	Model 1	Model 2	Model 3	Model 4
						HR (95% CI)			
Deaths (all cause)	Low decreasing	733	494	8402.2	58.8	Referent	Referent	Referent	Referent
	Light stable	1649	869	22 216.5	39.1	**0.73(0.65 to 0.81)**	**0.78(0.69 to 0.87)**	**0.80(0.71 to 0.90)**	**0.83(0.74 to 0.94)**
	Moderate increasing	849	372	12 034.9	30.9	**0.64(0.55 to 0.73)**	**0.71(0.61 to 0.81)**	**0.74(0.64 to 0.85)**	**0.76(0.66 to 0.88)**
	p trend					<0.001	<0.001	<0.001	<0.001
CVD mortality*	Low decreasing	733	198	8402.2	23.6	Referent	Referent	Referent	Referent
	Light stable	1649	297	22 216.5	13.4	**0.64(0.53 to 0.77)**	**0.67(0.56 to 0.81)**	**0.69(0.57 to 0.84)**	**0.76(0.62 to 0.91)**
	Moderate increasing	849	115	12 034.9	9.6	**0.53(0.42 to 0.67)**	**0.58(0.45 to 0.74)**	**0.61(0.48 to 0.78)**	**0.64(0.50 to 0.82)**
	p trend					<0.001	<0.001	<0.001	<0.001
All CVD events (fatal +non-fatal)†	Low decreasing	733	252	7901.3	31.9	Referent	Referent	Referent	Referent
	Light stable	1649	470	20 692.5	22.7	**0.80(0.69 to 0.94)**	**0.83(0.70 to 0.97)**	0.85 (0.72 to 1.00)	0.89 (0.75 to 1.04)
	Moderate increasing	849	198	11 338.1	17.5	**0.70(0.57 to 0.85)**	**0.74(0.60 to 0.90)**	**0.77(0.63 to 0.94)**	**0.78(0.64 to 0.95)**
	p trend					<0.001	0.002	0.009	0.015
CHD events (fatal+non-fatal)‡	Low decreasing	733	146	8190.7	17.8	Referent	Referent	Referent	Referent
	Light stable	1649	245	21 531.7	11.4	**0.73(0.59 to 0.91)**	**0.75(0.60 to 0.93)**	**0.77(0.62 to 0.95)**	**0.80(0.64 to 1.00)**
	Moderate increasing	849	98	11 672.4	8.4	**0.63(0.49 to 0.83)**	**0.67(0.51 to 0.88)**	**0.70(0.53 to 0.92)**	**0.71(0.54 to 0.93)**
	p trend					0.001	0.004	0.009	0.012
Stroke events (fatal+non-fatal)§	Low decreasing	733	76	8098.6	9.4	Referent	Referent	Referent	Referent
	Light stable	1649	186	21 308.6	8.7	0.99 (0.75 to 1.31)	1.04 (0.79 to 1.38)	1.08 (0.82 to 1.43)	1.10 (0.83 to 1.46)
	Moderate increasing	849	84	11 687.7	7.2	0.87 (0.63 to 1.20)	0.92 (0.66 to 1.28)	0.96 (0.69 to 1.34)	0.98 (0.70 to 1.37)
	p trend					0.368	0.573	0.773	0.851

Model 1, adjusted for age, occupational class, marital status, alcohol consumption, smoking status, region, previous diagnosis of MI, stroke or diabetes.

Model 2, Model 1+LDL, HDL, systolic blood pressure, insulin, waist circumference and FEV_1_.

Model 3, Model 2+IL-6 and vWf.

Model 4, Model 3+Hs TnT and NT-proBNP.

Boldface indicates statistical significance (p<0.05).

*Fatal CVD was defined as ICD-9 codes 390–459.

†All CVD events included all fatal CVD (ICD-9 codes 390–459) and non-fatal MI and stroke as described above.

‡Fatal myocardial infarction (MI) was defined as ICD-9 codes 410–414. Non-fatal MI was defined as heart attack or coronary thrombosis, in accordance with the WHO diagnostic criteria.

§Fatal stroke was defined as ICD-9 codes 430–438. Non-fatal stroke events included those that caused a neurological deficit for >24 hours.

CHD, coronary heart disease; CVD, stroke/MI; MI, myocardial infarction.

### Additional analyses

In several models, the proportional hazards assumption was not met for age. Where violated, age was collapsed into 10 categories and centred. Models were run again with the categorised age variable, but no meaningful differences were observed (data not shown). All associations were similar after removing men with pre-existing CVD (see online supplementary table S3) and the first 2 years of follow-up (data not shown).

#### Observed trajectories based on binary exposure measurements

44% (n=1312) of men were classified as persistently active (1-1-1), while membership of the other groups ranged from 5% to 13%. Lower unadjusted mortality rates were observed for the more active trajectories (see online supplementary table S4). The highest unadjusted all-cause mortality rate was found in men who had become inactive at the 20-year follow-up (1-1-0) (55.7 per 1000 person-years) which was comparable with the persistently inactive group (0-0-0) (51.5 per 1000 person-years). Compared with men who were persistently inactive, trajectory groups containing men who were active at the 20-year follow-up with various combinations of activity/inactivity at previous time points were associated with a lower risk of all-cause mortality in minimally and fully adjusted models. Similar risk reductions were observed in those who became active by the 20-year follow-up (0-0-1) and those who were persistently active (1-1-1). Persistent activity (0-1-1 and 1-1-1) appeared to be important for CVD mortality and CVD events, but there was still weak evidence to suggest that becoming active (0-0-1) can protect against CVD mortality.

## Discussion

Patterns of PA from midlife to old age were associated with all-cause and CVD mortality and major CHD and CVD events but not major stroke events. A moderate-increasing pattern was associated with the lowest risk of the aforementioned outcomes, but a light-stable pattern was also associated with significant benefit, which may be more achievable in the most inactive. Furthermore, adjustment for a range of confounders and mediating risk factors only partially explained these associations, suggesting that there may be other underlying biological pathways involved. For instance, physical activity may improve endothelial function and fibrinolytic activity; it may also have a direct effect on the cardiovascular system.^35^

It is well established that higher levels of PA are associated with a lower risk of mortality^1–4^ and our findings are consistent with this. Comparisons with previous studies investigating change in PA are not straightforward given the different methodologies used to identify trajectories. Studies reporting change across two time points suggest that initiating an active lifestyle can induce comparable survival benefits as those who are persistently active.^4 14 16 18 36^ Similarly, when using the observed trajectories based on binary exposure measurements, we found that current/most recent PA was critical in the protection against mortality. Indeed, those classified with increasing PA (0-0-1) had a reduced mortality risk similar to those who were persistently active (1-1-1). Although most survival benefits appear to be driven by most recent/current PA, engaging earlier in life is critical for continuation in old age when the potential benefits come to fruition. Shifting inactive adults to achieve modest amounts of PA in midlife or earlier may lead to a carry-over effect resulting in ongoing PA and long-term survival benefits.

Using GBTM, we did not identify groups of older adults that made meaningful changes in PA, highlighting that adults shifting from low to even modest levels of PA are rare. Instead, the GBTM trajectories suggest that prior PA dictates PA in old age. This is consistent with some other GBTM studies using continuous measures of PA that have also identified trajectories that do not typically overlap during this life period.^25 37 38^ In contrast, a recent study among retired adults using retrospectively reported PA showed that trajectories are more changeable across the life course than suggested by the present findings.^26^ Further research of this kind could help understand patterns of behaviour and inform interventions.

Studies using data-driven methods to identify PA trajectories and examine associations with subsequent mortality are scarce. One such study also identified three PA trajectories over a 7-year follow-up in an older sample.^25^ Similarly, these trajectories were largely determined by initial PA and the two most active trajectories were associated with a lower risk of all-cause and CVD mortality when compared with the least active. However, after adjusting for the final PA measure these associations were attenuated, suggesting that final PA was driving associations. The present study demonstrates similar findings but examines PA over a longer period and adjusts for a range of confounding and mediating factors. Notably, established risk factors only partially explained associations of PA trajectories with mortality and CVD. These associations also operated through inflammatory, haemostatic and cardiac markers, which is consistent with other studies in middle-aged^39–41^ and older adults.^20^ Despite the observed associations of PA trajectories with all-cause mortality, CVD mortality, CHD events and all CVD events, no associations were observed with stroke events when fatal and non-fatal were combined. Evidence linking PA and stroke has been mixed^42^ and the optimal type and amount of PA remains unclear. This study was consistent with studies reporting no association between PA and major stroke events.^43 44^ This may be explained by the lack of association observed between trajectory groups and blood pressure, which is an important risk factor for stroke, accounting for 62% of stroke events.^45^ However, further exploratory analyses revealed a significant risk reduction for a light-stable trajectory when only fatal stroke was considered. This is in agreement with previous findings from the BRHS that showed similar associations with a single measure of walking time.^46^ Few studies investigating life course or change in PA have examined associations with CVD subtypes or differentiated between fatal and non-fatal stroke. One such study in a sample of Californian women showed that the inverse association observed between PA and risk of stroke was largely driven by fatal stroke events.^47^ Parallels can also be drawn between this finding and those of other observational studies that have shown that modest levels of PA may provide the greatest protection from stroke.^43 47–49^ Strengths of this study include the prolonged follow-up, allowing the relationship between long-term PA trajectories and subsequent events to be examined. Other strengths include adjustment for a range of confounders and mediators and use of the same PA questionnaire at each wave. The GBTM approach allows the most naturally occurring trajectories to be identified, providing more accurate estimates of PA volume over time. An important limitation of this study is that subject attrition may have led to a biassed sample. The final sample was younger, healthier and more active than those who were excluded and may not be representative of the general population. This also further restricted the sample size, which may not have been sufficiently powered to detect associations with outcomes that have few events, such as stroke. Although the PA score has been validated against heart rate, FEV_1_ and device-measured PA, self-reports may still be subject to recall and social desirability bias.^31^ While we control for a range of confounding factors, residual confounding may be present. Unmeasured health problems may lead to inactivity and so the possibility of reverse causation remains, even after removing the first 2 years of follow-up. In addition, blood biomarkers are known to fluctuate, but they were measured at a single time point only. Furthermore, our sample comprises men, mainly of white British background and so our findings may not be generalisable to women and non-white ethnic groups.

## Conclusion

During the transition to old age, PA trajectories were associated with all-cause and CVD mortality and major CHD and CVD events. A dose–response relationship was observed, with a moderate-increasing pattern of PA providing the most protection against mortality and CVD, but even a sustained light level of PA was beneficial and may be more feasible for inactive adults.

**What is already known on this subject?**

- Higher levels of physical activity (PA) are associated with a reduced risk of all-cause mortality and major cardiovascular disease events in old age.
- There is some evidence that long-term changes in PA during adulthood can impact the risks of mortality and cardiovascular disease (CVD), but the conventional approaches used to define how PA changes may be inadequate for describing the true patterns of PA over time.

**What this study adds?**

- Moderate increasing levels of PA from midlife to old age were associated with the lowest risk of major coronary heart disease events, CVD mortality and all-cause mortality in later life.
- However, a more modest level of PA that is sustained across adulthood may be sufficient to reduce the risk of these outcomes in later life.
- Promoting a light sustained level of PA across adulthood may be a feasible intervention target for the most inactive and provides similar benefits as greater volumes of PA across the adult life course.
