# Supplementary material for: Trajectories of physical activity from midlife to old age and associations with subsequent cardiovascular disease and all-cause mortality
Source: J Epidemiol Community Health. 2019 Nov 8;74(2):130–6. doi: 10.1136/jech-2019-212706 (PMC6993021; doi:10.1136/jech-2019-212706)
Supplement: Supplementary data [file jech-2019-212706supp001.pdf]

Supplementary Text A

Cardiovascular Disease Biomarkers & Measures

A fasting blood sample was collected using the Sarstedt Monovette system. Total, HDL cholesterol and triglyceride were measured using a Hitachi 747 automated analyser (Hitachi, Tokyo, Japan). Total and HDL cholesterol were determined using methods described by Siedel et al., (18) and Sugiuchi et al., (19) and low-density lipoprotein was calculated using the Friedrickson-Friedwald equation. Serum insulin was measured using an enzyme-linked immunosorbent assay (ELISA) that does not cross-react with proinsulin.(20) Blood was anticoagulated with 0.109 mol/L trisodium citrate (9:1 vol:vol) for measurement of coagulation factor VIII in an MDA-180 coagulometer (Organon Teknika, Cambridge, UK). Plasma levels of von Willebrand factor were measured with ELISAs (DAKO, High Wycombe, UK). Interleukin-6 (IL-6) was assayed using a high-sensitivity ELISA (R&D Systems). N-terminal pro-brain natriuretic peptide was measured using the Elecsys 2010 (Roche Diagnostics, Burgess Hill, UK).(21) Cardiac troponin T was measured using a high-sensitivity method on an e411 analyser (Roche Diagnostics, Burgess Hill, UK).

Forced Expiratory Volume in one second (FEV_1) was measured using a Vitalograph Compact spirometer and was standardized for height by multiplying FEV1 by the square of the mean population height (meters) divided by each participant's height. Height was measured to the nearest 0.1cm using a stadiometer (Holtain, Crosswell, United Kingdom). Weight was measured in light clothing to the last complete 0.1kg using a digital electronic scale (Soehnle-Waagen, Murrhardt, Germany). Waist circumference was taken from the midpoint between the iliac crest and lower ribs and was measured using an insertion tape (CMS Ltd, London, United Kingdom). Systolic and diastolic blood pressure were each measured twice with the subject seated, using a Dinamap 1846SX blood pressure recorder and the mean of two readings was used.

Table S1. Model Search Process for Physical Activity Trajectories (n=3,231), British Regional Heart Study, 1978-2000

Number of groups	BIC	Log Bayes Factor (2*ΔBIC)	Estimated group %	Actual group %	Posterior probability
			64.3	64.8	0.95
2	-21050.2		35.7	35.2	0.93
			24.8	22.9	0.82
			49.1	50.9	0.84
3	-20880.7	339.0	26.1	26.2	0.90
			15.1	12.7	0.81
			49.1	52.2	0.84
			28.7	28.0	0.86
4	-20859.1	43.2	7.2	7.1	0.84
			4.0	4.5	0.77
			16.9	13.6	0.73
			44.1	47.3	0.80
			28.1	27.9	0.85
5	-20923.2	-134.4	6.9	6.8	0.85

BIC, Bayesian information criterion.

Models adjusted for employment status and number of cardiovascular disease diagnoses as time-varying covariates, and occupational class, marital status, number of children, region, body mass index , arthritis, bronchitis, blood pressure, breathlessness, chest pain, smoking status, alcohol consumption and breakfast consumption at baseline.

Table S2. Determining the Highest Model Function of the 3 Physical Activity Trajectory Groups (n=3,231), British Regional Heart Study, 1978-2000 [ab]

	1st iteration		2nd iteration			
**Group**	**Highest function**	***p***	**Highest function**	***p***	**Final estimated group %**	**Final actual group %**
Group 1	Quadratic	<0.001	Quadratic	<0.001	24.5	22.7
Group 2	Quadratic	0.937	Linear	0.019	49.3	51.0
Group 3	Quadratic	0.173	Linear	<0.001	26.2	26.3

[a] Starting with quadratic, the level of the polynomial function for each group was reduced at each iteration until each parameter estimate was statistically significant ($p<0.05$). [b] Models adjusted for employment status and number of cardiovascular disease diagnoses as time-varying covariates, and occupational class, marital status, number of children, region, body mass index, arthritis, bronchitis, blood pressure, breathlessness, chest pain, smoking status, alcohol consumption and breakfast consumption at baseline.

Table S3. Prospective association between GBTM-derived physical activity trajectory groups (from baseline [1978/80] to 20-year follow up [1998/2000]) and all-cause/cause-specific mortality and CVD events from 1998/2000 to 2016 in men without pre-existing CVD (n=2922)

Outcome	Trajectory group	N	N.o. of events	Person-years	Mortality/1000 person-years	Model 1	Model 2	Model 3	Model 4
						Hazard Ratio (95% CI)			
Deaths (all cause)	Low decreasing	1126	686	13945.6	49.2	Referent	Referent	Referent	Referent
	Light stable	1191	575	16573.4	34.7	**0.82 (0.73, 0.91)**	**0.86 (0.77, 0.97)**	**0.88 (0.78, 0.99)**	0.90 (0.81, 1.01)
	Moderate increasing	605	252	8784.7	28.7	**0.70 (0.60, 0.81)**	**0.76 (0.65, 0.88)**	**0.77 (0.67, 0.90)**	**0.81 (0.70, 0.95)**
	p trend					<0.001	<0.001	0.001	0.005
CVD mortality[a]	Low decreasing	1126	240	13945.6	17.2	Referent	Referent	Referent	Referent
	Light stable	1191	173	16573.4	10.4	**0.73 (0.60, 0.89)**	**0.75 (0.61, 0.91)**	**0.76 (0.62, 0.93)**	**0.80 (0.65, 0.98)**
	Moderate increasing	605	73	8784.7	8.3	**0.60 (0.46, 0.79)**	**0.59 (0.47, 0.76)**	**0.66 (0.50, 0.79)**	**0.73 (0.56, 0.96)**
	p trend					<0.001	<0.001	0.001	0.010
All CVD events (fatal + non-fatal)[b]	Low decreasing	1126	347	13015.9	26.7	Referent	Referent	Referent	Referent
	Light stable	1191	283	15604.0	18.1	**0.78 (0.67, 0.92)**	**0.80 (0.68, 0.94)**	**0.81 (0.69, 0.95)**	**0.83 (0.71, 0.98)**
	Moderate increasing	605	132	8347.1	15.8	**0.71 (0.58, 0.87)**	**0.74 (0.60, 0.91)**	**0.75 (0.61, 0.93)**	**0.80 (0.65, 0.99)**
	p trend					<0.001	0.001	0.003	0.016
CHD events (fatal + non-fatal)[c]	Low decreasing	1126	144	13553.0	10.6	Referent	Referent	Referent	Referent

	Light stable	1191	92	16102.1	5.7	**0.67 (0.51, 0.88)**	**0.69 (0.53, 0.90)**	**0.70 (0.53, 0.91)**	**0.74 (0.56, 0.97)**
	Moderate increasing	605	37	8563.6	4.3	**0.54 (0.37, 0.78)**	**0.58 (0.40, 0.84)**	**0.59 (0.40, 0.85)**	**0.66 (0.45, 0.96)**
	p trend					<0.001	0.001	0.001	0.011
Stroke events (fatal + non-fatal)[d]	Low decreasing	1126	124	13379.6	9.3	Referent	Referent	Referent	Referent
	Light stable	1191	111	16039.6	6.9	0.81 (0.62, 1.05)	0.82 (0.63, 1.07)	0.83 (0.64, 1.09)	0.85 (0.65, 1.10)
	Moderate increasing	605	58	8564.0	6.8	0.81 (0.58, 1.11)	0.82 (0.59, 1.14)	0.84 (0.61, 1.17)	0.87 (0.62, 1.20)
	p trend					0.126	0.172	0.221	0.301

Abbreviations: GBTM, group-based trajectory modelling; MI, myocardial infarction; CVD, stroke/MI

[a] Fatal CVD was defined as ICD-9 codes 390–459.

[b] All CVD events included all fatal CVD (ICD-9 codes 390–459) and non-fatal MI and stroke events.

[c] Fatal myocardial infarction (MI) was defined as ICD-9 codes 410–414. Non-fatal MI was defined as heart attack or coronary thrombosis, in accordance with the World Health Organisation diagnostic criteria.

[d] Fatal stroke was defined as ICD-9 codes 430–438. Non-fatal stroke events included those that caused a neurological deficit for >24 hours.

Model 1, adjusted for age, occupational class, marital status, alcohol consumption, smoking status, region, previous diagnosis of MI, stroke or diabetes

Model 2, Model 1 + LDL, HDL, systolic blood pressure, insulin, waist circumference and FEV_1

Model 3, Model 2 + IL-6 and vWf

Model 4, Model 3 + Hs-TnT and NT-proBNP

Table S4. Prospective association between manually-derived physical activity trajectory groups (from baseline [1978/80] to 20-year follow up [1998/2000]) and all-cause/cause-specific mortality and CVD events from 1998/2000 to 2016 (n=2998)

Outcome	Trajectory group*	N	N.o. of events	Person-years	Mortality/1000 person-years	Model 1	Model 2	Model 3	Model 4
						Hazard Ratio (95% CI)			
Deaths (all cause)	0-0-0	315	197	3829.2	51.5	Referent	Referent	Referent	Referent
	1-0-0	207	122	2555.2	47.8	1.03 (0.82, 1.29)	1.03 (0.82, 1.30)	1.03 (0.82, 1.29)	0.99 (0.79, 1.24)
	0-1-0	185	115	2118.5	54.3	0.97 (0.77, 1.22)	0.96 (0.76, 1.21)	0.97 (0.77, 1.23)	0.96 (0.76, 1.21)
	1-1-0	265	173	3104.1	55.7	1.12 (0.91, 1.38)	1.17 (0.95, 1.44)	1.16 (0.94, 1.42)	1.09 (0.89, 1.34)
	0-0-1	151	75	2089.5	35.9	**0.69 (0.53, 0.90)**	**0.71 (0.54, 0.93)**	**0.73 (0.56, 0.96)**	**0.71 (0.54, 0.93)**
	1-0-1	180	78	2537.8	30.7	0.79 (0.61, 1.03)	0.80 (0.61, 1.04)	0.85 (0.65, 1.11)	0.81 (0.62, 1.06)
	0-1-1	380	205	5165.6	39.7	**0.72 (0.59, 0.88)**	**0.78 (0.64, 0.95)**	**0.79 (0.64, 0.96)**	**0.79 (0.65, 0.97)**
	1-1-1	1315	615	18469.8	33.3	**0.72 (0.61, 0.84)**	**0.77 (0.65, 0.91)**	**0.78 (0.66, 0.93)**	**0.79 (0.67, 0.93)**
	p trend					<0.001	<0.001	<0.001	<0.001

CVD mortality[a]	0-0-0	315	84	3829.2	21.9	Referent	Referent	Referent	Referent
	1-0-0	207	42	2555.2	16.4	0.84 (0.58, 1.22)	0.86 (0.59, 1.25)	0.84 (0.58, 1.22)	0.77 (0.53, 1.12)
	0-1-0	185	48	2118.5	22.7	0.94 (0.66, 1.35)	0.97 (0.68, 1.39)	0.98 (0.69, 1.41)	0.99 (0.69, 1.42)
	1-1-0	265	57	3104.1	18.4	0.83 (0.59, 1.17)	0.87 (0.61, 1.22)	0.84 (0.59, 1.19)	0.75 (0.53, 1.06)
	0-0-1	151	31	2089.5	14.8	0.69 (0.45, 1.04)	0.71 (0.47, 1.07)	0.71 (0.47, 1.08)	0.67 (0.44, 1.02)
	1-0-1	180	27	2537.8	10.6	0.71 (0.46, 1.09)	0.70 (0.45, 1.09)	0.74 (0.48, 1.15)	0.70 (0.45, 1.09)
	0-1-1	380	65	5165.6	12.6	**0.54 (0.39, 0.75)**	**0.57 (0.41, 0.79)**	**0.57 (0.41, 0.79)**	**0.58 (0.42, 0.82)**
	1-1-1	1315	205	18469.8	11.1	**0.59 (0.46, 0.77)**	**0.64 (0.49, 0.84)**	**0.65 (0.50, 0.85)**	**0.65 (0.50, 0.85)**
	p trend					<0.001	<0.001	<0.001	<0.001
All CVD events (fatal + non-fatal)[b]	0-0-0	315	104	3629.2	28.7	Referent	Referent	Referent	Referent
	1-0-0	207	64	2363.2	27.1	1.09 (0.80, 1.50)	1.13 (0.83, 1.54)	1.12 (0.82, 1.53)	1.04 (0.76, 1.42)
	0-1-0	185	66	1988.8	33.2	1.12 (0.82, 1.52)	1.16 (0.85, 1.59)	1.18 (0.86, 1.61)	1.15 (0.84, 1.57)
	1-1-0	265	88	2856.7	30.8	1.10 (0.83, 1.47)	1.15 (0.86, 1.53)	1.12 (0.84, 1.50)	1.01 (0.76, 1.35)
	0-0-1	151	46	1940.6	23.7	0.89 (0.62, 1.26)	0.92 (0.65, 1.31)	0.93 (0.66, 1.33)	0.90 (0.64, 1.28)

	1-0-1	180	50	2294.3	21.8	1.04 (0.74, 1.46)	1.05 (0.74, 1.48)	1.10 (0.78, 1.55)	1.02 (0.72, 1.43)
	0-1-1	380	103	4840.8	21.3	0.76 (0.58, 1.00)	0.78 (0.59, 1.03)	0.79 (0.60, 1.04)	0.78 (0.59, 1.03)
	1-1-1	1315	326	17421.3	18.7	**0.79 (0.63, 0.99)**	0.83 (0.66, 1.05)	0.85 (0.68, 1.07)	0.82 (0.65, 1.03)
	p trend					<0.001	0.001	0.003	0.004
CHD events (fatal + non-fatal)[c]	0-0-0	315	58	3722.6	15.6	Referent	Referent	Referent	Referent
	1-0-0	207	38	2452.6	15.5	1.15 (0.76, 1.73)	1.18 (0.78, 1.78)	1.17 (0.78, 1.77	1.08 (0.72, 1.64)
	0-1-0	185	34	2074.6	16.4	1.03 (0.67, 1.57)	1.10 (0.72, 1.69)	1.11 (0.72, 1.70)	1.08 (0.70, 1.65)
	1-1-0	265	49	2999.6	16.3	1.12 (0.76, 1.64)	1.16 (0.79, 1.71)	1.14 (0.77, 1.68)	1.03 (0.70, 1.52)
	0-0-1	151	26	2035.4	12.8	0.89 (0.56, 1.42)	0.93 (0.59, 1.49)	0.94 (0.59, 1.51)	0.92 (0.57, 1.46)
	1-0-1	180	28	2396.4	11.7	1.08 (0.68, 1.70)	1.05 (0.67, 1.66)	1.09 (0.69, 1.72)	1.00 (0.63, 1.58)
	0-1-1	380	59	5002.5	11.8	0.81 (0.56, 1.16)	0.84 (0.58, 1.22)	0.85 (0.58, 1.23)	0.84 (0.58, 1.22)
	1-1-1	1315	167	17963.1	9.3	0.75 (0.55, 1.02)	0.81 (0.59, 1.10)	0.82 (0.60, 1.11)	0.79 (0.58, 1.08)
	p trend					0.003	0.012	0.017	0.020
Stroke events (fatal + non-fatal)[d]	0-0-0	315	34	3721.6	9.1	Referent	Referent	Referent	Referent

	1-0-0	207	18	2454.1	7.3	0.91 (0.52, 1.62)	0.95 (0.54, 1.69)	0.94 (0.53, 1.67)	0.92 (0.52, 1.62)
	0-1-0	185	20	2032.8	9.8	1.04 (0.60, 1.82)	1.06 (0.61, 1.85)	1.06 (0.61, 1.86)	1.04 (0.60, 1.82)
	1-1-0	265	33	2961.2	11.1	1.19 (0.73, 1.93)	1.25 (0.77, 2.04)	1.23 (0.75, 2.00)	1.16 (0.71, 1.89)
	0-0-1	151	17	1994.7	8.5	0.96 (0.54, 1.73)	1.00 (0.56, 1.80)	1.02 (0.56, 1.83)	1.00 (0.56, 1.80)
	1-0-1	180	20	2404.3	8.3	1.08 (0.61, 1.88)	1.08 (0.62, 1.90)	1.15 (0.66, 2.02)	1.10 (0.63, 1.93)
	0-1-1	380	39	4996.9	7.8	0.82 (0.51, 1.30)	0.85 (0.53, 1.36)	0.86 (0.53, 1.37)	0.85 (0.53, 1.36)
	1-1-1	1315	130	17901.4	7.3	0.88 (0.59, 1.29)	0.92 (0.62, 1.36)	0.94 (0.64, 1.39)	0.93 (0.63, 1.37)
	p trend					0.229	0.344	0.431	0.465

Abbreviations: MI, myocardial infarction; CVD, stroke/MI

* Physical activity trajectories correspond to levels of physical activity from baseline through 12 and 20 year follow ups. 1 indicates at least 'light' physical activity levels and 0 denotes 'inactive' or 'occasional' activity levels. For example, (0-0-0) represents low physical activity at all periods, whilst (1-0-0) indicates high physical activity at baseline only.

[a] Fatal CVD was defined as ICD-9 codes 390–459.

[b] All CVD events included all fatal CVD (ICD-9 codes 390–459) and non-fatal MI and stroke events.

[c] Fatal myocardial infarction (MI) was defined as ICD-9 codes 410–414. Non-fatal MI was defined as heart attack or coronary thrombosis, in accordance with the World Health Organisation diagnostic criteria.

[d] Fatal stroke was defined as ICD-9 codes 430–438. Non-fatal stroke events included those that caused a neurological deficit for >24 hours.

Model 1, adjusted for age, occupational class, marital status, alcohol consumption, smoking status, region, previous diagnosis of MI, stroke or diabetes

Model 2, Model 1 + LDL, HDL, systolic blood pressure, insulin, waist circumference and FEV_1

Model 3, Model 2 + IL-6 and vWf

Model 4, Model 3 + Hs-TnT and NT-proBNP
